# A service evaluation of outcomes in two in-patient recovery units

**DOI:** 10.1192/pb.bp.116.055137

**Published:** 2017-12

**Authors:** Rob Macpherson, Claudia Calciu, Chris Foy, Kim Humby, Dave Lozynskyj, Charles Garton, Hannah Steer, Helen Elliott

**Affiliations:** 1 Gether Foundation NHS Trust; 2 Gloucestershire Hospitals NHS Foundation Trust

## Abstract

**Aims and method** To evaluate outcomes for patients during their admission or in the first year of treatment in two in-patient recovery units. Changes in health and social functioning, service use and need (rated by patients and staff) were evaluated.

**Results** In 43 patients treated, there was a large (30%) increase in patients discharged to their own tenancies, rather than supported accommodation. There was minimal change in Health of the Nation Outcome Scales (HoNOS) scores in the course of the admission but staff- and patient-rated unmet needs reduced and met needs increased. Needs changed mainly in domains relating to social functioning. Reductions in risk to self and others were rated by staff but not patients. There were no cases of patients being readmitted to acute hospital during the study period.

**Clinical implications** Although these results offer some support to the treatment approach described in these in-patient recovery units, further research in larger samples is needed to identify how these services can best be deployed to help individuals with severe mental illness and complex needs.

From the start of 2011 it was decided to develop greater understanding of the outcomes of the work of our two 2gether Foundation National Health Service (NHS) Trust recovery in-patient units, by evaluating outcomes assessed by staff and patients at admission, mid-point and discharge from the units. These services have in the past decade adopted a strong recovery-based model of practice, in line with the UK government policy *No Health Without Mental Health*.^1^ A major strategic aim in this policy is for more people with mental health problems to recover, defined as: ‘a greater ability to manage their own lives, stronger social relationships, a greater sense of purpose, the skills they need for living and working, improved chances in education, better employment rates and a suitable and stable place to live’.^1^ The literature on personal recovery emphasises the individual journey in recovery, in which regaining hope, taking back control and finding new opportunities are key processes.^2^ It has been argued that a transformation of mental health services is required for this to occur;^3^ mental health professionals recognising patients' strengths and expertise as experts in their own health and acknowledging the importance of personal narratives, alongside other forms of evidence. The increasing focus in UK and international healthcare policy on recovery has occurred at a time of generally increasing personal autonomy in wider society while health services have shifted their focus towards helping every individual to make the most of their health, rather than relating mainly to those with illness.^4^

The recovery units studied provide active rehabilitation for 10 and 13 residents respectively. The units are remote from the main psychiatric hospital services but provide full in-patient support and are registered to take detained patients. The focus of the work in the units is with patients who have complex, severe mental illness, predominantly psychosis, characterised by treatment resistance and other complications. Treatment is provided through a full multi-disciplinary team and includes approaches to optimise health through use of medication, talking therapies including family work, guidance on healthy living, and treatment and support for substance use problems. A wide range of social approaches include help with daily living skills including budgeting, self-care, shopping and cooking, help with finances, benefits and accommodation, and support to develop interests and activity such as sport, leisure activities and work. The work in these units differs from acute in-patient work in focusing more on treating long-term, disabling mental health problems and impaired social functioning over longer time periods, rather than dealing with brief, episodic illness. Typical admissions last approximately 9 months, rather than just over 1 month in acute units (based on November 2016 2gether Foundation NHS Trust data).

The original model of the recovery units described was developed to help patients in their rehabilitation from long-stay hospitals,^5^ aiming to support patients with complex illness to reintegrate into the community. They have subsequently evolved to work mainly with patients who need longer-term rehabilitation after acute psychiatric admission or any patients who have developed disability due to severe mental illness. Over the past 20 years in the UK, in association with NHS and local authority funding changes, there has been a disinvestment in these services,^6^ despite recognition in recent commissioning guidance^7^ that this form of treatment is needed as part of a spectrum of care provision in each district.

The evidence from research in this field has previously been reviewed,^8^ showing that in-patient recovery units can effectively support most patients accepted by them, the majority of whom can be resettled to less dependent community placements over a period of months or years. The research indicates that treatment in recovery units is associated with improvement in social functioning and social networks and reduced levels of negative symptoms in schizophrenia. Placements are associated with relatively few long-term readmissions. These findings have been replicated in other countries such as Australia, the USA Northern Ireland, Norway and Italy.^9^ The quality of the research evidence is however variable and there has been very little formal research carried out in this area.^5^ A recent study of outcomes 5 years after in-patient rehabilitation^10^ found that around 40% of patients had remained in stable accommodation or moved to independent placement and sustained this.

Although UK commissioning frameworks have consistently argued the need for services of this type to help patients with complex needs in their pathway from acute or secure hospital to the community, there still seems to be a shortage of appropriate residential places in some areas, particularly for those with the most severe and enduring mental health problems. A study of hospital services in Birmingham, UK,^11^ showed that long-stay (more than 6 months) patients were consistently found to occupy 20% of acute beds. The authors noted that where staff made recommendations for community placements, ‘by far the majority’ required 24-hour residential care, suggesting a need to improve access to this sort of provision. A recent paper^12^ noted that problems may arise from the fragmentation due to use of private sector psychiatry and made a strong case for local, well-organised, NHS rehabilitation services. A survey^13^ of English rehabilitation services showed that despite closures of nearly all NHS long-stay hospital beds, most areas still had active rehabilitation units available to help people with complex, treatment-resistant illness. In 93 local authority regions, most (77%) had short-term (up to 12 months) rehabilitation units, with an average of 13 beds. It appears that although services are available in most areas, the level of provision is variable and patchy. Hospital services in the UK continue to be under pressure and there are often suggestions of a need for more beds. However, it has been argued^14^ that alternatives to acute in-patient admission could reduce this pressure and that around one in four cases in acute units could be managed in a unit run by nurses or care workers.

## Aims of the evaluation

This service evaluation aimed to describe, prospectively, aggregated outcomes for new residents over the duration of their admission or in the first year of treatment. The evaluation considered changes in health and social circumstances from admission to the units, to discharge or 1 year after admission. Utilisation of acute hospital treatment was monitored before and during treatment in the recovery units.

## Method

This project was carried out as a service evaluation with a formal protocol and a project steering group which met regularly during the project's work. The County Research and Development Support Unit assessed and gave written agreement for the project, prior to commencement. The use of aggregated data was preferred to other methods, such as the use of within-participant findings. This was a pragmatic decision, as the introduction of routine outcome monitoring was hoped to improve and make more systematic individual care planning, as well as to enable individual and service-level evaluation of outcome. The measures used in the project were selected to support the new way of working.

### Participants

At the point of admission to the unit, demographic and health-related data were collected for all patients, on a specially designed form. The patient's key worker rated the Camberwell Assessment of Need Short Appraisal Schedule (CANSAS)^15^ and Health of the Nation Outcome Scales (HoNOS)^16^ scores at the time of admission. CANSAS forms were given by key workers to patients for self-completion, with an explanation about how to complete them. Key workers were instructed to ensure that patient rating of need was independent of their own rating and access to support from local advocacy services was offered if this was considered helpful. Where a carer had a significant role with the patient, this individual was also approached to assess the needs of the patient (with consent, following the usual clinical permissions pathway).

To be included in the evaluation, patients were required to have continuous treatment for a minimum of 3 months in one of the units. This was to ensure that patients who were occasionally admitted briefly from the acute ward, due largely to problems such as homelessness leading to delays in discharge, but were not assessed to require the therapeutic approach of the recovery unit, did not bias the sample. It was also felt that patients discharged before 3 months would not have had time to engage meaningfully with the therapeutic work of these services.

The same ratings were repeated 6 months after admission and at discharge, or at the end of the first year of treatment. Demographic and health-related data were collected at the point of discharge from the unit or at the end of the first year of admission.

### Measures

The CANSAS^15^ rates need as ‘absent’ (no problem), ‘met’ (problem addressed by services) or ‘unmet’ (significant, ongoing problem) across 22 social and healthcare domains. The HoNOS^16^ is a 12-item scale that rates various aspects of health and social functioning on a 5-point Likert scale to measure levels of problem severity.

### Data management

At admission, patients were allocated a number for identification purposes and from this time all data were held anonymously, with the identification number only used on forms, held securely by the unit administration lead, who managed the data collection processes. Data sheets were held in a locked office in secure filing cabinets or on a password-protected Trust PC.

### Statistical analysis

C.F. advised on the use of descriptive, demographic and illness-related information and the analysis of change scores from admission to discharge using aggregated HoNOS scores and CANSAS total, met and unmet need total scores. Data were entered into SPSS for analysis (SPSS version 18 for Windows). Non-parametric Wilcoxon matched pairs signed ranks tests were used to assess changes in mean HoNOS and CANSAS ratings.

### Ethical consideration

This work was an attempt to evaluate whether the recovery units were effective in their intended work. It was a service evaluation and did not have a randomised controlled design. Information was collected as part of the work of the units and it was used at an individual level to more systematically understand patient need and plan treatment. There was no intention to use experimental tools or to compare units or employ a control group. As a result of the design it was noted that results would not be generalisable, although it was hoped that they may be of value at a time of greater attention to treatment outcomes and considering issues of service quality and innovation. Patient consent was not formally taken but was considered to be given by participation, where patients gave individual ratings of need and this was always explained to be optional and unrelated to the rest of the patient's treatment. Prior to starting the project, the need for formal ethics committee submission was considered by our County Research Support Unit, which advised that this was not required and gave written approval for the project.

## Results

Data collection was carried out in the two units from April 2011 to June 2014. During that time a total of 43 patients were admitted for a period of at least 3 months. Data were collected on the patients over this period, the gaps in data being largely due to the challenges of managing data collection within a standard clinical setting, rather than (with the infrastructure permitted by research funding) as a research project. Gaps in the data-set are reported within the results presented below. These related often to patient choice and willingness to participate in routine data collection. Although access to advocacy was freely available and advocates were working regularly in both units through the project, we do not know how often they provided specific support to patients in completing outcome measures. We believe this was rare and in the great majority of cases patients completed forms independently or with minor support from staff.

The study group of 43 patients included 28 (65%) male patients, aged 18–62 years (mean 36.5, s.d. = 10.3); 39 (91%) were single, 2 (5%) married and 2 (5%) divorced. Most patients (*n* = 23, 53%) were admitted from an acute psychiatric ward, the remainder (*n* = 20, 47%) direct from the community. In the year prior to the recovery unit admission, patients had spent a mean of 20.7 weeks (range 0–52, s.d. = 24.5) in a psychiatric hospital. Patients had experienced a mean of 1.0 admissions in the year before entering the recovery unit (range 0–4, s.d. = 0.55).

At the time of admission all patients were unemployed, and 23 (53%) lived in supported accommodation, 19 (44%) in their own tenancy and 1 (2%) with family. Overall, 27 (63%) were under an assertive outreach team, 11 (26%) under a community recovery team (community mental health team) and 5 (12%) under early intervention services.

Patients spent a mean of 380 days (s.d. = 177) in the recovery units; there were no recorded episodes of acute psychiatric readmission during this time. In total, 38 of discharges (74%) were planned and 5 patients (12%) were discharged for other reasons.

At the time of discharge, 42 (98%) were unemployed, 1 patient being a part-time student. Overall, 32 (74%) had their own tenancy, 4 (9%) were living in supported accommodation and 2 (5%) were living with family. In total, 29 (67%) were under an assertive outreach team, 11 (26%) under a community recovery team and 3 (7%) were under early intervention services.

The main changes over the course of this evaluation were: there was a small increase in employment following treatment in the units; there was a 30% increase in patients living in their own tenancy; and some patients were taken over by assertive outreach teams during their admission, mostly moving from early intervention teams.

No individuals were readmitted to acute in-patient care during their recovery in-patient admission.

Baseline and final mean HoNOS and CANSAS met/unmet need scores are presented in Table 1.

**Table 1:** Mean HoNOS and CANSAS ratings at baseline and discharge/12-month follow-up

	Baseline rating Mean (s.d.)	Discharge/12-month rating Mean (s.d.)	Wilcoxon signed ranks 2-tailed test
HoNOS	19.9 (8.2)	18.5 (9.0)	*Z*= −1.46, *P*>0.05

Staff-rated CANSAS met need	7.2 (4.3)	7.3 (3.9)	*Z*= −0.41, *P*>0.05

Staff-rated CANSAS unmet need	3.7 (3.6)	3.4 3.0)	*Z*= −0.76, *P*>0.05

Patient-rated CANSAS met need	3.9 (4.5)	4.8 (4.4)	*Z*= −1.39, *P*>0.05

Patient-rated CANSAS unmet need	2.8 (3.3)	2.2 (2.5)	*Z*= −1.32, *P*>0.05

HoNOS, Health of the Nation Outcome Scales; CANSAS, Camberwell Assessment of Need Short Appraisal Schedule.

CANSAS scores by domain at baseline and discharge/12 months are represented in Table 2 for patient ratings and Table 3 for staff ratings.

**Table 2:** Patient CANSAS ratings of met, unmet and no needs by domain at baseline and discharge/12 months

	Met need			Unmet need			No need			Total completed CANSAS ratings
	Baseline	12 months	Change (%)	Baseline	12 months	Change (%)	Baseline	12 months	Change (%)	
Social life	5	13	8 (28)	13	10	−3 (10)	11	6	−5 (17)	29

Psychological distress	7	12	5 (17)	10	6	−4 (13)	13	12	−1 (3)	30

Physical health	10	12	2 (7)	6	5	−1 (3)	15	14	−1 (3)	31

Intimate relationships	5	3	−2 (7)	9	14	5 (17)	16	13	−3 (10)	30

Daytime activities	10	16	6 (21)	10	4	−6 (21)	9	9	0 (0)	29

Sexual expression	3	4	1 (3)	10	10	0 (0)	16	15	−1 (3)	29

Accommodation	9	19	10 (33)	8	1	−7 (23)	13	11	−2 (7)	30

Psychotic symptoms	11	14	3 (10)	11	7	−4 (13)	9	7	−2 (7)	31

Safety to self	9	6	−3 (10)	4	2	−2 (7)	18	23	5 (16)	31

Information on treatment	18	23	5 (16)	2	2	0 (0)	11	6	−5 (16)	31

Enough food	14	18	4 (13)	2	3	1 (3)	15	10	−5 (16)	31

Use of public transport	7	3	−4 (13)	4	7	3 (10)	19	20	1 (3)	30

Basic education	2	8	6 (19)	2	0	−2 (7)	27	23	−4 (13)	31

Budgeting	8	8	0 (0)	5	10	5 (16)	18	13	−5 (16)	31

Safety to others	3	1	−2 (7)	0	1	1 (3)	27	28	1 (3)	30

Care of home	8	15	7 (23)	3	3	0 (0)	12	12	0 (0)	30

Self-care	11	10	−1 (3)	3	2	−1 (3)	17	19	2 (7)	31

Non-prescribed drugs	4	2	−2 (7)	2	1	−1 (3)	25	28	3 (10)	31

Benefits taken up	9	10	1 (4)	1	4	3 (12)	16	12	−4 (15)	26

Use of telephone	4	5	1 (3)	0	0	0 (0)	27	26	−1 (3)	31

Alcohol problems	2	4	2 (7)	4	1	−3 (10)	25	26	1 (3)	31

Childcare	2	1	−1 (3)	1	0	−1 (3)	27	29	2 (7)	30

CANSAS, Camberwell Assessment of Need Short Appraisal Schedule.

**Table 3:** Staff CANSAS ratings of met, unmet and no needs by domain at baseline and discharge/12 months

	Met need			Unmet need			No need			Total completed CANSAS ratings
	Baseline	12 months	Change (%)	Baseline	12 months	Change (%)	Baseline	12 months	Change (%)	
Social life	12	18	6 (15)	18	16	−2 (5)	10	6	−4 (10)	40

Psychological distress	19	21	2 (5)	11	8	−3 (8)	8	9	1 (3)	38

Physical health	18	20	2 (5)	7	7	0 (0)	14	12	−2 (5)	39

Intimate relationships	7	3	−4 (13)	13	16	3 (9)	12	13	1 (3)	32

Daytime activities	22	23	1 (3)	16	13	−3 (8)	2	4	2 (5)	40

Sexual expression	8	3	−5 (18)	10	13	3 (11)	10	12	2 (7)	28

Accommodation	12	21	9 (23)	13	9	−4 (10)	15	10	−5 (13)	40

Psychotic symptoms	20	23	3 (8)	18	14	−4 (10)	1	2	1 (3)	39

Safety to self	19	10	−9 (23)	4	4	0 (0)	16	25	9 (23)	39

Information on treatment	27	33	6 (15)	2	0	−2 (5)	12	8	−4 (10)	41

Enough food	20	22	2 (5)	3	2	−1 (3)	17	16	−1 (3)	40

Use of public transport	7	4	−3 (8)	6	7	1 (3)	24	26	2 (5)	37

Basic education	7	11	4 (10)	1	0	−1 (2)	33	30	−3 (7)	41

Budgeting	17	13	−4 (10)	9	13	4 (10)	13	13	0 (0)	39

Safety to others	17	7	−10 (25)	2	2	0 (0)	21	31	10 (25)	40

Care of home	11	15	4 (11)	12	10	−2 (6)	13	11	−2 (6)	36

Self-care	19	17	−2 (5)	6	7	1 (2)	16	17	1 (2)	41

Non-prescribed drugs	14	5	−9 (23)	2	3	1 (3)	24	32	8 (20)	40

Benefits taken up	20	22	2 (6)	1	0	−1 (3)	13	12	−1 (3)	34

Use of telephone	5	7	2 (5)	0	0	0 (0)	36	34	−2 (5)	41

Alcohol problems	15	13	−2 (5)	6	4	−2 (5)	20	24	4 (10)	41

Childcare	3	4	1 (3)	1	1	0 (0)	35	34	−1 (3)	39

CANSAS, Camberwell Assessment of Need Short Appraisal Schedule.

There were improvements in patient-rated met needs in domains relating to accommodation, social life, care of the home and daytime activity, most other domains showing no change or minor increases and decreases. There were reductions in patient-rated unmet needs in accommodation, daytime activity budgeting and intimate relationships, with about half the domains showing minimal change. It can be seen that the main changes in patient-rated need were in domains relating to social functioning.

There were increases in staff-rated met needs in domains relating to accommodation, social life and information about treatment. Apparent reductions in staff-rated met need in the domains safety to others, safety to self and use of non-prescribed drugs appeared to be explained by comparable increases in levels of ‘no need’ in these domains. Staff-rated unmet needs showed smaller levels of change, the domains which reduced most being accommodation, daytime activities and psychotic symptoms.

## Discussion

We have shown that it is possible as part of routine clinical outcome measurement to assess longitudinal outcomes in a standard recovery in-patient setting, using staff- and patient-rated measures. The results included some gaps in data which were due largely to patients declining to complete CANSAS ratings of need or incomplete participation by staff members. However, the results seem likely to be reasonably representative of the patients admitted to the units over this time, other than those patients who were admitted and then discharged or readmitted to the acute ward within a short time (these patients were excluded from the study population).

### Results and limitations of research

We found minimal change in HoNOS scores in the course of the recovery unit admission but overall staff and patient assessed unmet needs tended to reduce and met needs tended to increase, although not at a statistically significant level. This may have related to the relatively small sample size. The main changes in need found over the study period rated by staff and patients related to improved social functioning, a finding which accords with the primary clinical aims of these services; to help individuals to regain life skills lost through periods of severe illness and ideally to try to achieve the most independent living situation possible. Our evaluation included patient and staff evaluations of need, both being included as the research indicates that they differ, and that the patient's perspective may be particularly important.^17^ The reduction in staff-assessed risk to self and others was encouraging and may have linked to reduced substance misuse, as these units have a strict drug-free policy and during admission patients are supported to remain drug free. A recent survey^18^ of in-patient rehabilitation units in Birmingham, UK, found chronically high levels of problematic and socially inappropriate behaviours and suggested that new approaches, focusing on engagement and the management of challenging behaviour, may be helpful.

A number of limitations result from the method of this service evaluation. There was no control group and, as a result, the findings cannot be generalised. We are only aware of the existence of one, small, randomised controlled trial in this area,^19^ and arguably this type of research is not really feasible in this setting,^5^ although it remains vital to learn more about outcomes in these important and relatively expensive services. The numbers in our study group were small and it would be helpful to compare our results with studies using similar outcome measures in other settings, ideally with larger patient groups. Further limitations arise from the lack of data relating to progress at different time points during the treatment in the recovery units: it is possible that greater benefits occur early or later in the treatment process, which we were unable to evaluate. The scales used allow limited understanding of the patient experience, which could be more fully accessed through the use of qualitative studies and a number of tools assessing aspects of patient-rated recovery are now available.^20^ Qualitative studies in this area have emphasised the importance of choice and autonomy for many patients^21^ and have shown the potential for personal recovery to be facilitated through appropriate supported living accommodation.^22^

Of interest, poorer outcomes were associated with non-adherence to medication and our finding that most patients were discharged to assertive outreach teams suggests that many patients will continue to need a high level of support following treatment in recovery units. A recent study using retrospective care records^9^ found significantly reduced hospital admission 2 years after in-patient rehabilitation and that a substantial proportion of the sample went into more independent living. We found a large increase (30%) in the number of patients discharged to their own tenancies, rather than supported accommodation.

### Consideration of findings against previous research

At a time of increasing pressure on in-patient services, our findings accord with previous research^23^ suggesting that alternatives to acute in-patient care could reduce this pressure and that many cases could be managed in facilities such as recovery units. More than half the admissions to our in-patient recovery units were from acute wards and their ability to manage cases effectively without return to hospital and, most importantly, achieve clinical improvements, emphasises the importance of having these services available in each region/district where acute units operate. Our findings were in line with previous research^14^ showing improved outcomes in terms of accommodation and stable social functioning following treatment in recovery units. It is important to have a long-term perspective due to the ongoing, high levels of chronic morbidity in this population.^10^ There is a continuing need for different types of supported accommodation, although in our study a substantial number progressed to fully independent living.

Research in supported accommodation has been classified^5^ into three domains: quality of care; external evaluation and quality of life; and subjective satisfaction by the resident. In terms of quality of care, the most important factors appear to be the effectiveness of an individually centred, targeted programme of care and the quality of the physical environment. A systematic review of the quality of care in longer-term mental healthcare settings^23^ found eight domains of institutional care that were key to recovery: living conditions, interventions for schizophrenia, physical health, restraint and seclusion, staff training and support, therapeutic relationship, autonomy and patient involvement. The two units investigated have both embraced the recovery approach, working alongside patients in pursuit of their goals and promoting autonomy and empowerment of the individual. Previous research^24^ has shown that quality of care is heavily determined by the personality and orientation of project leaders and staff working in these units drew on extensive experience of local rehabilitation/recovery work, which has resulted in three major reviews and redevelopment of services and extensive staff training in recovery methods. The units were both accredited as ‘excellent’ in 2016 within the rehab-AIMS national benchmarking of rehabilitation units.^25^ However, the results in our study show that the patient population is highly disabled with high levels of need and high usage of hospital care. Our results, showing clinical stability alongside progress in personal and social domains, was encouraging and hopeful. These units support many patients who due to their illness have difficulty creating their own structure, with consequent loss of internal security and often associated fears of losing control. The provision of support, treatment and therapeutic approaches available 24 hours provides an important support^26^ which enables patients to feel stable, secure and then able to progress with rebuilding their lives. It is however important to consider differences between professional and patient preferences. When asked their view, patients have tended to prefer the option of their own, independent accommodation^27^ over rehabilitation or supported accommodation. Family members tend to align with the professional view and prefer their relatives being cared for in staffed environments.^28^ An important criticism of staffed settings is the potential for institutional regimes and a poor rehabilitative culture, which could impede independence and autonomy.^29^ Conversely, some patients and family members have reported that independent tenancies can be socially isolating^27^ and it seems that many patients benefit from treatment in these units, particularly if they maintain a collaborative approach and provide a wide range of therapeutic options.
